# Interventions for anxiety and depression in patients with atopic dermatitis: a systematic review and meta-analysis

**DOI:** 10.1038/s41598-024-59162-9

**Published:** 2024-04-17

**Authors:** Stella P. Hartono, Sheena Chatrath, Ozge N Aktas, Stephanie A Kubala, Korey Capozza, Ian A. Myles, Jonathan I. Silverberg, Alan Schwartz

**Affiliations:** 1 Laboratory of Allergic Diseases, National Institute of Allergy and Infectious Diseases, 10 Center Drive, Bethesda, MD 20852 USA; 2 University of Illinois College of Medicine, Peoria, IL USA; 3 Global Parents for Eczema Research, Santa Barbara, CA USA; 4 Laboratory of Clinical Immunology and Microbiology, National Institute of Allergy and Infectious Diseases, Bethesda, MD USA; 5 Department of Dermatology, George Washington University School of Medicine and Health Sciences, Washington, DC USA; 6 University of Illinois at Chicago, Chicago, IL USA

**Keywords:** Atopic dermatitis, Eczema, Anxiety, Depression, Quality of life, Skin diseases

## Abstract

Atopic dermatitis (AD) is a chronic inflammatory skin disease that is associated with anxiety and depression. Few studies have addressed interventions for symptoms of anxiety and depression in this population. To determine the efficacy of interventions for anxiety and depression in patients with AD. PubMed, MEDLINE, EMBASE, and PsycINFO were searched from inception to November 2023. English-language studies published in peer-reviewed journals evaluating the effect of interventions on anxiety and/or depression using validated assessment tools on patients with AD were included. Titles, abstracts, and articles were screened by at least two independent reviewers. Of 1410 references that resulted in the initial search, 17 studies were included. Fourteen of these studies are randomized controlled trials, while the other 3 studies are prospective controlled trials with pre and post-test designs. Data were extracted using a standardized extraction form, and the Preferred Reporting Items for Systematic Reviews and Meta-analyses (PRISMA) guidelines were followed. To accommodate trials with multiple interventions (each compared to a control group), we conducted a mixed-effects meta-analysis with the trial as a random effect. Prespecified outcomes were changes in symptoms of anxiety and depression in patients with AD as evaluated using standardized assessment tools. Of the 17 studies included in this systematic review, 7 pharmacological intervention studies with 4723 participants examining 5 different medications were included in a meta-analysis. Of these studies, only 1 study evaluated medications prescribed to treat anxiety and/or depression; the rest evaluated medications prescribed to treat AD. Meta-analysis of all the pharmacological interventions resulted in significant improvement in anxiety, depression, and combined anxiety-depression scale scores (standardized mean difference [95% CI]: − 0.29 [− 0.49 to − 0.09], − 0.27 [− 0.45 to − 0.08], − 0.27 [− 0.45 to − 0.08]) respectively. The 10 non-pharmacological studies with 2058 participants showed general improvement in anxiety but not depression. A meta-analysis of the non-pharmacological interventions was not conducted due to variable approaches and limited data. Pharmacological interventions designed to improve AD were found to improve anxiety and depression in patients with moderate-severe disease. More comprehensive studies on non-pharmacological and pharmacological interventions that primarily target anxiety and depression are needed.

## Introduction

Atopic dermatitis (AD) is a chronic inflammatory skin disease associated with profound physical and mental health symptoms^1–4^. There is a significantly higher prevalence of clinical depression (10.1% vs. 4.3%), anxiety disorder (17.2% vs. 11.1%), and suicidal ideation (12.7% vs. 8.3%) among patients with common skin diseases such as AD compared to general population^5^. Over time, adult patients tend to have fluctuating levels of depression that can wax and wane with AD symptoms. However, certain depressive symptoms, such as thoughts of self-harm, feeling bad, difficulty concentrating, and slow movement are persistent over time despite improvement in AD severity^6^. Additionally, adults with AD have significant impairments in their quality of life. Many patients report avoidance of social gatherings and activities, and loss of productivity at work or missing work due to their AD^7–9^. In the pediatric population, patients with severe AD symptoms have increased internalizing behaviors and depressive symptoms^10^. Moreover, caregivers, particularly those caring for children with severe AD, commonly experience symptoms of anxiety and depression^11–13^.

Although the mental health burden in patients with AD and their caregivers was well studied, few studies addressed interventions for patients with AD experiencing symptoms of anxiety and depression. Previous studies suggested that controlling AD severity may improve depressive symptoms in AD. However, some patients may have more persistent depressive symptoms and may need additional therapies or interventions^6^. In this paper, we review pharmacological and non-pharmacological interventions for patients experiencing symptoms of anxiety or depression.

## Methods

### Literature search and study selection

This study was conducted in accordance with the Preferred Reporting Items for Systematic Reviews and Meta-Analyses (PRISMA) guidelines^14^. We conducted a systematic literature review search of interventional studies published through November 2023 in PubMed, MEDLINE, EMBASE, and PsycINFO using MESH or EMTREE terms “atopic dermatitis” or “eczema” and “anxiety” or “depression” (see Supplementary Table 1 for search details). In addition, reference lists of identified reviews and book chapters were also reviewed for additional studies. Unpublished manuscripts, conference proceedings/abstracts, case reports, and dissertations were not included. Title and abstract screening were conducted by two independent reviewers, with discrepancies resolved by discussion and consensus. A subsequent full-text review was conducted by four independent reviewers, and the decision to include or exclude the study was made by discussion and consensus.

### Inclusion and exclusion criteria

We included all studies that met the following criteria: (a) published in English in a peer-reviewed journal, (b) participants were diagnosed with AD, (c) anxiety and/or depression were evaluated using validated assessment tools, (d) complete data (baseline data and end-point data) was accessible for analysis, and (e) sample size of the study was greater than or equal to 10 participants for each treatment arms to ensure the studies reviewed were adequately powered. Studies that measured health-related quality of life (HRQOL) were evaluated and included if anxiety or depression was part of the HRQOL assessment tool utilized. Both adult and pediatric studies are included in this review, and the findings are organized as pharmacological vs. non-pharmacological interventions. The complete review protocol has not been registered in advance and is available upon request.

### Data extraction and quality assessment

We used a standardized extraction form to extract data, including reference of study, publication year, study design, patient characteristics, number of participating subjects, intervention, and outcome measures. The primary outcome we evaluated was a change in the level of anxiety and/or depression from baseline post-intervention. As multiple results may be available for different time points within the same study arms in the study reports, we used the following rules to extract data from each study. When possible, we extracted outcome data for anxiety and depression under each treatment arm to allow us to evaluate various dosages and treatment frequencies for pharmacological interventions. Some studies reported combined data for anxiety and depression, and these outcomes were analyzed separately. If data were available at multiple time points within the trial, we only used the data at the final time point of each intervention for meta-analysis. The risk of bias was assessed by evaluating the blinding of participants and personnel, allocation concealment, selective reporting, and incomplete outcome data using RoB2^15^ and ROBINS-I^16^. Studies excluded due to small number of participants were also summarized to minimize potential bias.

### Statistical analysis

Where data permitted, we planned to characterize the pooled effects from randomized controlled trials (RCT) of pharmacological or non-pharmacological treatments through meta-analyses. We performed meta-analyses using R 4.2 (R Core Team, Vienna, Austria) and the *metafor* package^17^. To accommodate trials with multiple interventions (each compared to a control group), we conducted mixed-effects meta-analysis with trial as a random-effect. We also fitted three-level models with intervention nested within the trial and unstructured covariance matrices to represent trials that compared several interventions to a common control group, but these models produced statistically indistinguishable results. Accordingly, we report the results of the simpler model. We estimated the pooled standardized mean difference (SMD) in outcomes (anxiety, depression, or combined anxiety-depression scale scores), in order to combine scores from different instruments and report results on a common effect size measure. We assessed heterogeneity using Cochran’s Q test. We examined funnel plots for publication bias and conducted meta-regressions to examine the impact of drug across all trials and the impact of dosage in Dupilumab trials. Due to the paucity of data from studies with non-pharmacological treatments, we were unable to perform meta-analyses and performed a narrative summary instead.

## Results

### Search results

**Figure 1:**
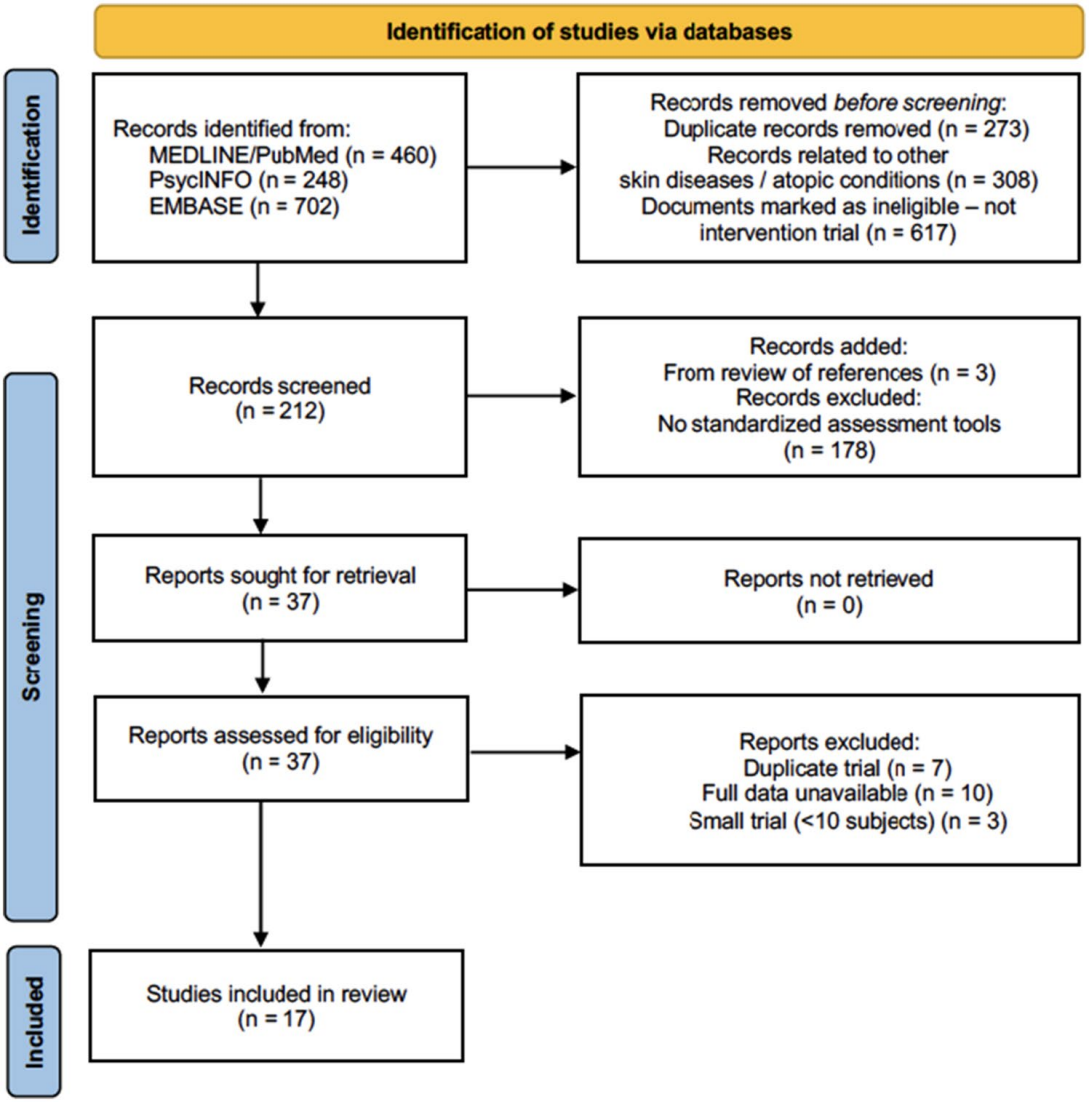
PRISMA diagram of the study selection for this systematic review. Seventeen studies are included in this systematic review. Seven pharmaceutical interventions out of the seventeen studies are included in the meta-analysis.

Our searches retrieved 1410 references, of which 212 were interventional studies (Fig. 1). Among these, we identified 37 studies evaluating the effects of pharmacological and non-pharmacological interventions on anxiety and/or depression in both adults and pediatric patients with AD. Two of the non-pharmacological interventions evaluated anxiety and depression in the caregiver^18,19^. Primary corresponding authors of these studies were contacted twice when data was not readily available from the manuscripts. Seven studies were excluded due to duplicated primary data^20–26^, while 10 studies were excluded due to data unavailability^27–36^. Three studies^37–39^ were excluded due to a small sample size (< 10 subjects), resulting in 17 final studies.

### Study characteristics

#### Pharmacological intervention

There are 7 pharmacological interventions identified by our systematic review (Table 1,^40–46^). Two of the pharmacological intervention studies were phase 2b RCT and five of the studies were phase 3 RCT. Studies conducted by Kawana, et al. were sponsored by a government grant while the rest of the RCTs were industry sponsored. Only one study evaluated the treatment of anxiety and/or depression using an anxiolytic/antidepressant (Tandospirone citrate (TC), which is a partial agonist of the 5-HT1A receptor and is more commonly used in China and Japan for the treatment of patients with anxiety disorders^46^. The six remaining studies are interventions to treat moderate-severe AD (Abrocitinib, Baricitinib, Dupilumab, Tralokinumab). All studies were conducted in adults; our literature search did not identify any pediatric pharmacological interventions.

**Table 1:** Pharmacological intervention included in the meta-analysis.

Author group (Ref)	Year	Study design	Drug evaluated	Study arms	Study length (weeks)	Study population	Sample Size (total)	Assess-ments used	Outcome
Thyssen et al.^40^	2022	RCT	Abrocitinib and Dupilumab	Abrocitinib 100 mg q1d	16	Adults	837	HADS	● Anxiety and depression significantly reduced in all treatment arms ● Abrocitinib 200 mg daily showed greatest reduction
				Abrocitinib 200 mg q1d					
				Dupilumab 300 mg q2w					
				Placebo					
Thyssen et al.^41^	2022	RCT	Baricitinib	Baracitinib 1 mg q1d	16	Adults	1568	HADS	● Anxiety improved in all treatment arms compared to placebo ● Depression improved with Baricitinib 2 mg, 4 mg, and combination 4 mg + TCS group
				Baracitinib 2 mg q1d					
				Baracitinib 4 mg q1d					
				Baracitinib 2 mg q1d + TCS					
				Baracitinib 4 mg q1d + TCS					
				Placebo					
Silverberg et al.^42^	2021	RCT	Tralokinumab (TK)	TK 45 mg q2w	12	Adults	204	MCS	Improved the mental component summary at 12 weeks compared to placebo
				TK 150 mg q2w					
				TK 300 mg q2w					
				Placebo					
DeBruin-Weller et al.^43^	2018	RCT	Dupilumab	Dupilumab 300 mg q1w + TCS	16	Adults	318	HADS	HADS < 8 was achieved in the q2week group but was not achieved in the q1 week group
				Dupilumab 300 mg q2w + TCS					
				Placebo + TCS					
Simpson et al.^44^	2016	RCT	Dupilumab	Dupilumab 300 mg q1w	16	Adults	1379	HADS	Anxiety and depression significantly reduced in all treatment arms
				Dupilumab 300 mg q2w					
				Placebo					
Simpson et al.^45^	2016	RCT	Dupilumab	Dupilumab 300 mg q1w	16	Adults	380	HADS	● Anxiety and depression significantly reduced at all doses and frequency ● 300 mg doses have the greatest efficacy in reducing anxiety and depression symptoms
				Dupilumab 300 mg q2w					
				Dupilumab 300 mg q4w					
				Dupilumab 100 mg q4w					
				Dupilumab 200 mg q4w					
				Placebo					
Kawana et al.^46^	2010	RCT	Tandospirone citrate (TC)	TC 30 mg q1d	4	Adults	37	POMS	Significant improvement in anxiety, depression, stress, and insomnia
				Untreated					

*RCT* Randomized control trial, *TCS* topical corticosteroids, *HADS* Hospital Anxiety and Depression Scale (0–21 scale), *POMS* Profile of Mood States, *MCS* Mental component summary-summarized vitality, social functioning, role-emotional, and mental health domains.

The Profile of Mood States score (POMS)^47^, the Hospital Anxiety and Depression scale (HADS)^48^, and Short Form 36-Mental Component Summary (SF36v-MCS)^49^ were used to evaluate anxiety and depression outcomes. The POMS questionnaire assesses six mood subscales: tension-anxiety, depression, anger-hostility, vigor, fatigue, and confusion. Higher scores in these subscales excluding vigor indicate greater levels of anxiety and depression. HADS is a self-reported scale including 14 items (7 items each for anxiety and depression). A total subscale score of > 8 points out of possible 21 for each subscale indicates greater levels of anxiety and depression. SF36 is comprised of 36 questions and 9 domains that assess physical and mental health. The MCS domain assesses mental health domains, social function, vitality, and emotional health. The change in severity score for anxiety and depression is provided in Table 2.

**Table 2:** The change in severity score for pharmaceutical intervention.

Author group (Year)	Study length (weeks)	Sample size (group)	Study arms	Anxiety	Depression
Thyssen et al.^40^	16	238	Abrocitinib 100 mg q1d	− 1.2 (− 1.6, -0.8)	-1.0 (-1.4, -0.7)
		226	Abrocitinib 200 mg q1d	− 2.0 (− 2.4, -1.6)	-1.6 (-1.9, -1.2)
		241	Dupilumab 300 mg q2w	− 1.5 (− 1.9, − 1.1)	− 1.2 (− 1.5, − 0.8)
		131	Placebo	− 0.4 (− 0.9, 0.1)	− 0.3 (− 0.8, 0.2)
Thyssen et al.^41^	16	88	Baracitinib 1 mg q1d	− 1.6 ± 0.8	− 1.5 ± 0.9
		78	Baracitinib 2 mg q1d	− 1.9 ± 0.7	− 2.7 ± 0.9
		82	Baracitinib 4 mg q1d	− 2.2 ± 0.7	− 2.8 ± 0.8
		60	Baracitinib 2 mg q1d + TCS	− 1 ± 0.6	− 2.1 ± 0.62
		59	Baracitinib 4 mg q1d + TCS	− 1.2 ± 0.52	− 2.3 ± 0.66
		162	Placebo	− 0.9 ± 0.48	− 0.3 ± 0.57
		44	Placebo + TCS	− 0.9 ± 0.58	− 1.3 ± 0.70
Silverberg et al.^42^	12	38	TK 45 mg q2w	− 1.95 ± 0.31	
		42	TK 150 mg q2w	− 1.77 ± 0.30	
		42	TK 300 mg q2w	1.96 ± 0.30	
		38	Placebo	− 0.71 ± 0.31	
DeBruin-Weller^42^	16	110	Dupilumab 300 mg q1w + TCS	− 5.2 ± 5.56	
		107	Dupilumab 300 mg q2w + TCS	− 6.1 ± 5.59	
		108	Placebo + TCS	− 2.3 ± 5.82	
Simpson et al.^44^	16	223	Dupilumab 300 mg q1w*	− 5.2 ± 7.48	
		224	Dupilumab 300 mg q2w*	− 5.2 ± 7.47	
		224	Placebo*	− 3 ± 4.19	
		239	Dupilumab 300 mg q1w**	− 5.8 ± 4.33	
		233	Dupilumab 300 mg q2w**	− 5.1 ± 4.27	
		236	Placebo**	− 3 ± 4.3	
Simpson et al.^45^	16	63	Dupilumab 300 mg q1w	− 2.2 ± 0.4	− 2.4 ± 0.4
		64	Dupilumab 300 mg q2w	− 2.2 ± 0.4	− 2.0 ± 0.4
		65	Dupilumab 300 mg q4w	− 1.3 ± 0.4	1.4 ± 0.4
		65	Dupilumab 100 mg q4w	− 1.4 ± 0.4	− 1.0 ± 0.5
		61	Dupilumab 200 mg q4w	− 1.9 ± 0.4	− 2.0 ± 0.5
		61	Placebo	− 0.4 ± 0.5	0.4 ± 0.5
Kawana et al.^46^	4	20	TC 30 mg q1d	− 2	− 4.5
		17	Untreated	0.8	0.2

##### Anxiety

**Figure 2:**
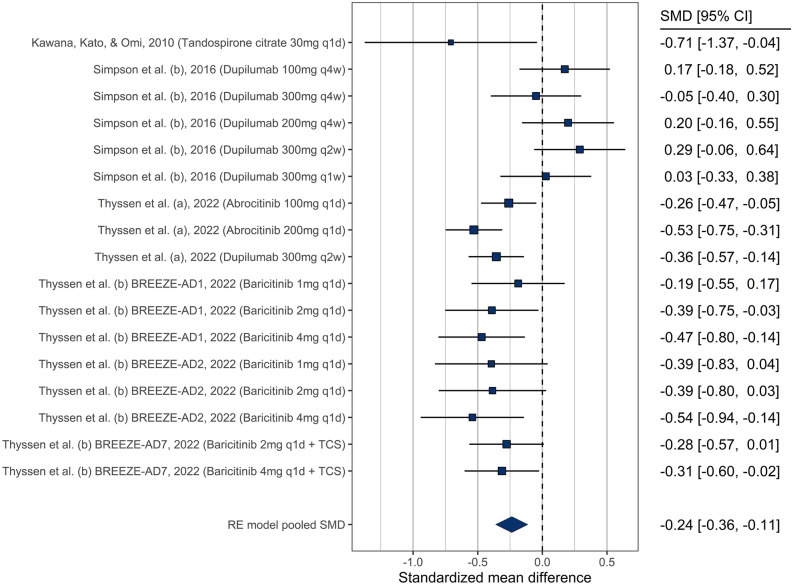
Forest plot showing treatment effectiveness of various study arms in anxiety.

Of the studies related to anxiety, 7 evaluated multiple doses of the intervention or dosing frequencies resulting in 17 comparisons. Eight of these comparisons showed a statistically significant decrease in anxiety at the end of the study compared to baseline (Fig. 2). The meta-analysis indicated decreased anxiety levels in patients receiving pharmacological therapy for their AD: SMD = − 0.29, 95% CI [− 0.49 to − 0.09], with significant heterogeneity (Q = 38.8, *p* = 0.001). The funnel plot did not suggest publication bias (Supplementary Fig. 1), and neither type of drug nor dosage (only available for dupilumab) was a significant moderator of the effect (*p* = 0.34 and *p* = 0.67, respectively) (supplementary Fig. 2 and 3).

##### Depression

**Figure 3:**
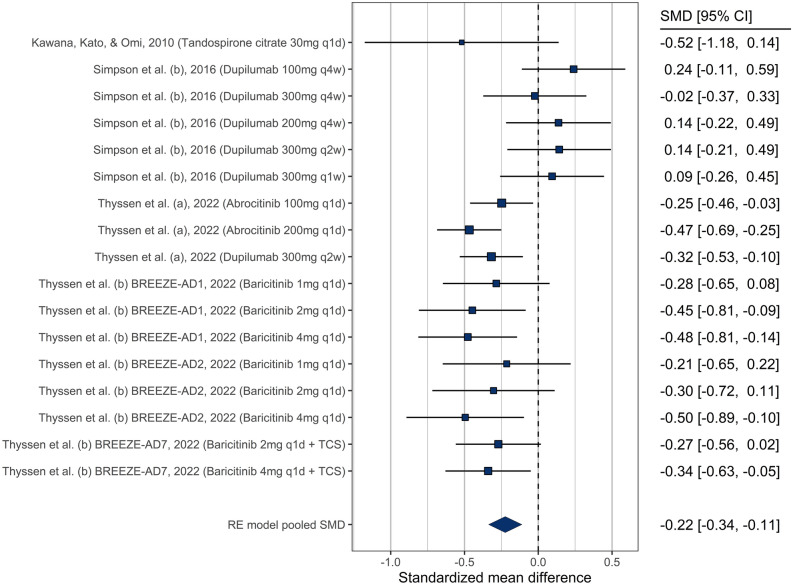
Forest plot showing treatment effectiveness of various study arms in depression.

Similarly to anxiety, 7 studies related to depression evaluated multiple dosing regimens resulting in 17 comparisons. Seven comparisons showed a statistically significant decrease in depression by the end of the study compared to baseline (Fig. 3, see Table 1 for individual summary of each study). Overall, there was an improvement in depression severity in patients receiving pharmacological interventions for AD (SMD = − 0.27 [− 0.45 to − 0.08]), with significant heterogeneity (Q = 31.8, *p* = 0.01). We observed no publication bias (supplementary Fig. 1), and no significant moderation by drug (*p* = 0.36) or dosage (*p* = 0.75) (supplementary Fig. 2 and 3).

**Figure 4:**
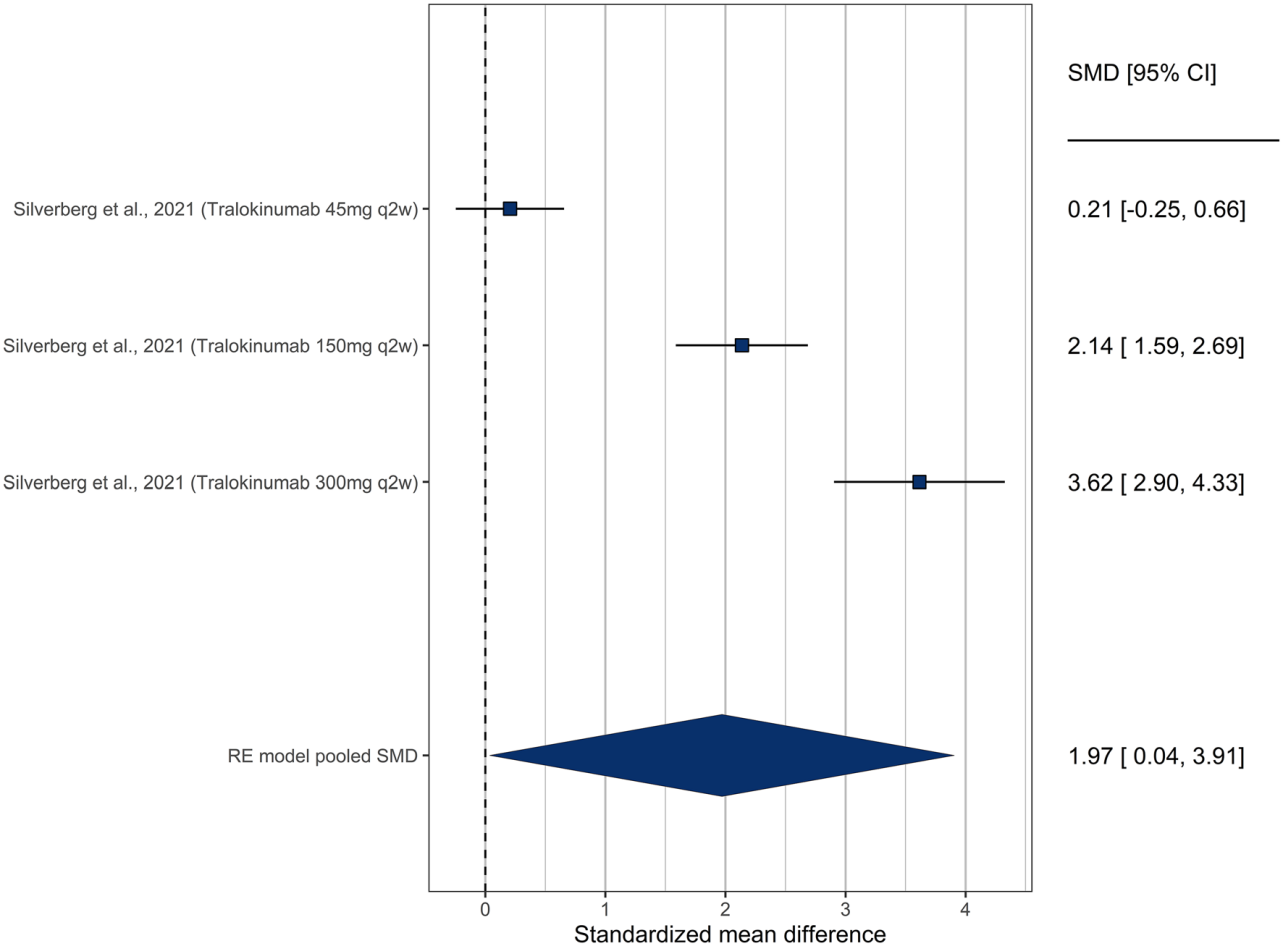
Forest plot showing treatment effectiveness of various study arms in Silverberg et al.^31,42^. This study was graphed separately as it used SF-36v MCS to assess emotional and mental health, which we consider a surrogate measurement for depression.

There was one study^42^ that did not specifically evaluate anxiety or depression, but rather assessed mental health domains using the SF36-MCS and should be considered as a surrogate measurement for depression. In this study, three unique dosages of Tralokinumab were evaluated. The 150 mg and 300 mg of tralokinumab showed significant improvement in MCS scores, while the 45 mg dosage did not. Overall, there was a significant improvement in MCS score (SMD = 1.49 [1.18–1.80]) (Fig. 4).

##### Combined anxiety and depression

**Figure 5:**
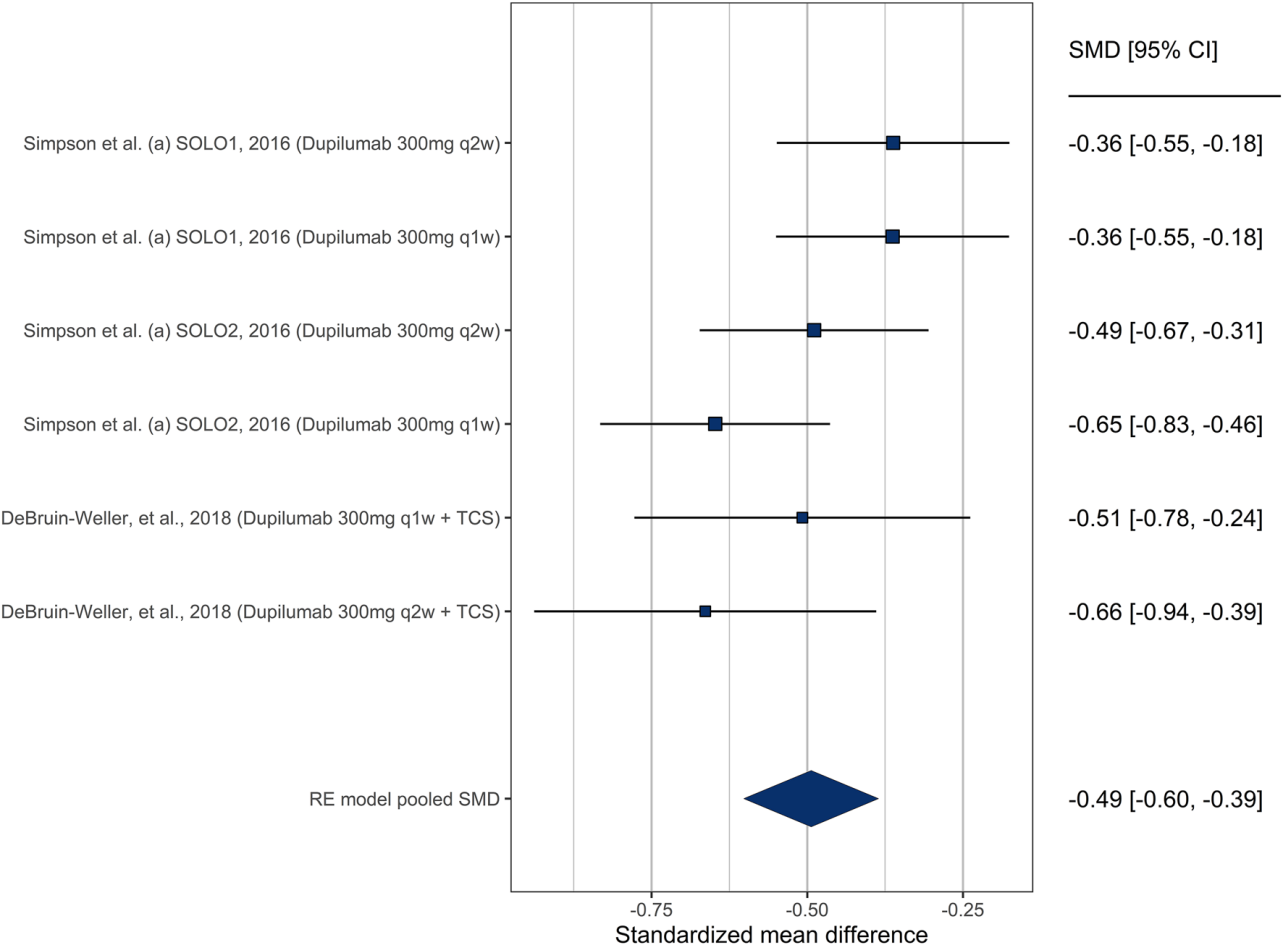
Forest plot showing treatment effectiveness of various study arms in both anxiety and depression combined.

The combined HADS score was also evaluated in studies that did not provide sub-scores for anxiety and depression. There were 6 comparisons evaluating the change in HADS score from prior to initiation of the drug and at the end of the study. All 6 comparisons showed a statistically significant improvement in HADS score separately (Fig. 5), leading to an overall significant improvement in HADS score (SMD = − 0.50 [− 0.064 to − 0.35]). Heterogeneity was not significant (Q = 7.9, *p* = 0.16), and we observed no publication bias (supplementary Fig. 1). As all these studies involved dupilumab, we did not test for moderation by drug due to lack of moderation by dosage of dupilumab (*p* = 0.66).

#### Non-pharmacological intervention

We identified 10 non-pharmacological interventions (Table 3,^18,19,50–57^). Of these, there were 7 RCT, 1 non-randomized controlled trial, 1 open-label pilot clinical trial, and 1 prospective cohort study with pre- and post-test design with no control group. There were 6 adult studies^18,19,51,52,56,57^, 2 pediatric studies^53,54^, and 2 combined studies in both adults and children^50,55^. Sample sizes of these studies were generally small with significantly variable interventions.

**Table 3:** Non-pharmacological intervention.

Author group (Ref)	Year	Study design	Intervention evaluated	Study length (weeks)	Study population	Sample size (total)	Assess-ments used	Outcome
Kishimoto et al.^57^	2023	RCT	Online mindfulness and self-compassion training	13	Adults	107	HADS	Significant improvement in anxiety (*p* < 0.001) and depression (*p* < 0.001) in the treatment group
Muzzolon et al.^53^	2021	controlled, non-RCT	Educational intervention	8–20	Pediatric (14–18 yo) and caregivers	48	CDLQI & DFIQ	Significant improvement in the anxiety measures in both children and caregivers
Hedman-Lagerlof et al.^51^	2019	Prospective, pre-and post-test design	Exposure based CBT	10	Adults	9	BAI & MADRS-S	● Significant improvements in general anxiety (*p* < 0.005) ● No significant improvement in depression
Bae et al.^50^	2012	RCT	Progressive muscle relaxation (PMR)	4	Adults and pediatrics	25	BDI & STAI	Significant improvement in depression and anxiety
Thomas et al.^54^	2011	RCT	Ion-Exchange Water Softeners	12	Pediatrics	336	DFIQ	Significant improvement in depressive measures in DFIQ
Guerra-Tapia et al.^55^	2007	RCT	Educational intervention (information leaflets)	24	Adults and pediatrics	1247	STAI	● Significant improvement in anxiety compared to baseline ● No significant improvement in anxiety compared to patients receiving standard treatment
Linnet et al.^52^	2001	RCT	Dynamic psychotherapy treatment	24	Adults	32	STAI	Significant improvement in anxiety only on patients with an initial high level of trait anxiety (TA)
Ehlers et al.^56^	1995	RCT	Dermatologic education, relaxation therapy, CBT, combination education and CBT (DECBT), and standard medical care	12	Adults	137	STAI & CES-D	● Significant reduction of anxiety after educational interventional program (both with and without behavioral therapy) ● No significant difference is observed for depression in all treatment group
Lin et al.^19^	2022	RCT	Infant massages	60	Adults (mothers of infants with AD)	97	SAS & SDS	Significant improvement in SAS and SDS in mothers (*p* < 0.01)
Yoo et al.^18^	2018	Prospective, pre-and post-test design	Educational session—in person and online	8	Adults (mothers of pediatric AD patients)	20	STAI	Significant improvement in anxiety at the end of the program (*p* < 0.001)

*CBT* cognitive behavioral therapy, *RCT* Randomized control trial, *BDI* Beck Depression Inventory, *STAI* State-Trait Anxiety Inventory, *BAI* Beck Anxiety Inventory, *MADRS-S* Montgomery Åsberg Depression Rating Scale Self-report, *CDLQI* Children's Dermatology Life Quality Index, *DFIQ* the Dermatitis Family Impact Questionnaire, *CES-D* Center for Epidemiological Studies Depression Scale, *SAS* self-rating anxiety scale, *SDS* self-rating depression scale.

The non-pharmacological interventions consisted of educational interventions, psychotherapy, infant massages, progressive muscle relaxation (PMR), and use of ion-exchange water softener for improving eczema severity. Anxiety and depression were measured using HADS, Beck Depression Inventory (BDI)^58^, State-Trait Anxiety Inventory (STAI)^59^, Montgomery-Asperg Depression Scale (MADRS)^60^, Dermatitis Family Impact Questionnaire (DFI)^61^, Center for Epidemiological Studies Depression Scale (CES-D)^62^, Self-rating Anxiety Scale (SAS)^63^ and Self-rating Depression Scale (SDS)^64^.

##### Anxiety

Four studies (40%) evaluated educational interventions. Educational interventions are delivered as instructional leaflets, face-to-face sessions, online learning, or a combination of both and range between 8 to 24 weeks in duration. Patients and caregivers who received the educational intervention showed significant improvement in anxiety, except for the study by Guerra-Tapia et al.^55^. Although patients in the interventional arms of this study showed significant improvement in their anxiety compared to baseline, this is not significantly different compared to the standard treatment group at any time point throughout the study. Of note, this was the only study where the educational material was given as an instructional leaflet instead of an in-person session.

Four studies (40%) evaluated psychotherapy interventions either online or through in-person sessions. Kishimoto et al. evaluated an integrated online group therapy program of self-compassion and mindfulness for 13 weeks^57^. Hedman-Lagerlof et al. evaluated CBT, where patients were taught to refrain from scratching upon exposure to stressful situations in addition to mindfulness training^51^. Linnet et al. evaluated psychological sessions with a trained psychologist for 6 months^52^. Ehlers et al. evaluated CBT aimed at reducing scratching frequency as well as increasing patients’ ability to cope with itching and stress^56^. Of these 4 studies, Ehlers et al. only observed significant improvement in anxiety when CBT was combined with education, while the rest reported significant improvement in anxiety with psychotherapy alone, especially in patients with initial high levels of trait anxiety^52^.

Three studies (30%) evaluated relaxation techniques. Bae et al.^50^ evaluated 4 weeks of PMR in both adults and children, Ehlers et al.^56^ evaluated relaxation training based on auto-suggestive sessions led by clinical psychologists, and Lin et al.^19^ evaluated the effectiveness of infant massages. Patients who received PMR showed significant improvement in anxiety, but although patients who received relaxation training showed improvement in anxiety over baseline, it was not significant compared to patients receiving standard topical treatment alone. Lin et al. showed decreased anxiety in mothers who were taught to give once daily infant massages with skin oil for about 10 min. Although it is difficult to objectively evaluate anxiety and depression in infants, it should be noted that infants who received the massage interventions showed significant improvement in their caregiver reported quality of life scores in this study.

One study evaluated the use of ion-exchange water softener, and this study did not result in significant improvement in anxiety. All three studies evaluating caregivers showed significant improvement in the anxiety measures with educational interventions^18,53^ and infant massages^19^.

##### Depression

Neither educational or psychotherapy interventions resulted in significant improvement in depressive symptoms, whereas infant massages, PMR, and the use of ion-exchange water softener resulted in improvement in depressive symptoms. Meta-analysis was not conducted due to limited data available from the studies.

### Excluded studies

We excluded three studies^37–39^ based on their small sample size. Cheirif-Wolosky et al. performed a retrospective analysis of children with AD between 2001 to 2018 who required systemic therapy for 3 months or longer^37^. Of the 21 children evaluated, 13 patients (61.9%) presented with anxiety and depression at baseline. Twelve patients received oral methotrexate, 9 received systemic steroids, 8 received oral thalidomide, 4 received azathioprine, 2 received mycophenolate mofetil, and 1 patient received cyclosporine. The duration of treatments ranged between 5.5 to over 20 months. As this is a retrospective study evaluating skin improvement as the primary outcome, anxiety and depression are not consistently evaluated across all treatment group. Wittkowski and Richards evaluated the benefits of 8-weeks cognitive behavioral therapy (CBT) in two adults with AD^38^. Both patients showed improvements in anxiety but only one patient showed improvement in depression. Modell et al. evaluated 3 weeks of Buproprion 150 mg/day and 300 mg/day dosages in an open-label study on 10 adult patients with AD and 10 adult patients with psoriasis (no placebo control arm) and showed skin improvement in 6 out of 10 AD patients (*p* = 0.0003) and 8 out of 10 patients with psoriasis (*p* = 0.001)^39^. They did not list any quantitative measures of anxiety and depression, which is also a criterion for exclusion in our meta-analysis.

### Quality of individual study and risk of bias

**Figure 6:**
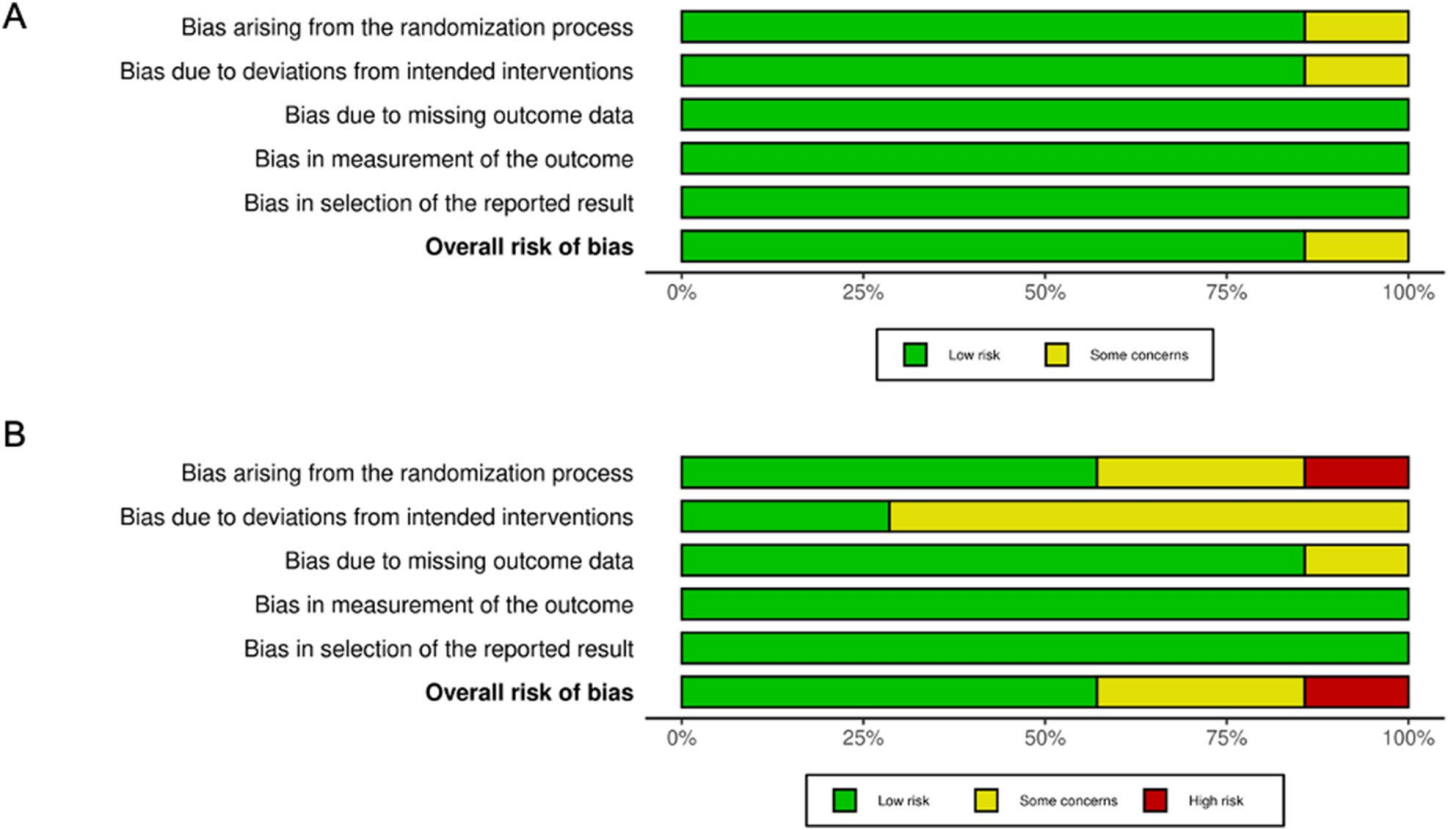
Risk of bias of the pharmaceutical (**A**) and non-pharmaceutical interventions (**B**) based on 5 different domains.

We assessed the risk of bias in individual studies using the Cochrane Risk of Bias tool^15,16^. There were generally fewer elements of high risk of bias in the newer studies compared to the older studies. As most of the pharmacological interventions evaluated were double blind RCT conducted after 2015, there is low concern for risk of bias in these studies (Fig. 6A). There are more elements with high risk of bias in the non-pharmacological interventions, associated with difficulty in blinding inherent in educational interventions and cognitive behavioral therapy treatment, as well as deviations from the intended protocol (Fig. 6B).

## Discussion

This is the first study to systematically review the impact of pharmacologic and non-pharmacologic interventions for AD on anxiety and depression symptoms in patients with AD. Patients with AD have increased levels of anxiety, depression, mental health hospitalizations, and increased suicidal ideations^2,3,65–67^. This mental health burden has been shown to be persistent over the course of the disease and is thought to wax and wane with the severity of AD symptoms^6^. Results from this systematic review indicate that although psychological interventions (both pharmacological and non-pharmacological) may play a key role in AD treatment, only few studies investigated the effects of such interventions in this patient population.

### Pharmaceutical interventions

Various studies have shown that severe AD, pruritus, skin pain, and facial involvement have higher associations with anxiety and depression^3,6,68^. Given the persistence of mental health symptoms and the chronicity of AD, adequate control of AD symptoms may be effective in improving mental health comorbidities and the quality of life for patients with AD. We found that patients receiving pharmacological interventions aimed to control their AD symptoms showed a decrease in their levels of anxiety and depression in conjunction with the improvement of their skin. These results support the previous suggestions that improvement of AD severity may reduce mental health symptoms^6^.

While the first-line treatment for anxiety and depression includes selective serotonin/norepinephrine reuptake inhibitors (SSRI/SNRI)^69^, few studies have evaluated the efficacy of these medications in patients with AD. There was only one study^46^ in this review that systematically evaluated the use of an anxiolytic/antidepressant as a treatment for anxiety and depression in patients with AD. In addition to improvement in symptoms of anxiety and depression, there was also a significant decrease in the SCORAD index of the treated group compared to the untreated group. The investigator proposed inhibition of stress-induced mast cell degranulation as a possible mechanism for this effect based on their prior observation in the mouse model. Several case reports and small studies have observed more rapid and/or pronounced improvements in skin symptoms when anxiolytic/antidepressants were used in conjunction with common dermatologic therapy^70–72^. This is in support of how emotional stress can lead to AD exacerbation^73,74^. Larger studies are needed to evaluate the efficacy of antidepressant therapies in patients with AD not only as a treatment for anxiety and depression but also to determine their utility as an adjunct therapy for AD.

Most of the studies we included in this meta-analysis are clinical trials of drugs with relatively recent FDA approval intended for patients with moderate-severe eczema, with anxiety and depression as a secondary outcome. There was no RCT that evaluated the efficacy of topical medications or systemic glucocorticoids in improving anxiety and depression in patients with AD. This reflects the recent increased awareness of anxiety and depression as a co-morbidity associated with AD. However, as trials included in this study only evaluated patients with moderate-severe eczema, further research is needed to understand whether pharmacological interventions are equally effective for patients without moderate-severe disease or patients who do not qualify for immunomodulating therapies.

### Non-pharmaceutical interventions

Compared to pharmacological interventions, there were fewer non-pharmacological interventional trials in the literature that addressed anxiety and depression in patients with AD. Interventions that incorporated in-person education and exposure-based CBT were effective in reducing anxiety in patients and caregivers as opposed to providing educational material alone, highlighting the necessity of interpersonal connection in the management of anxiety in patients with AD. Only one study^56^ did a head-to-head comparison between educational intervention, CBT, and relaxation techniques. Of these, only the educational program with or without CBT was shown to significantly improve anxiety.

Both education and exposure-based CBT did not show significant improvement in depression. Similar to our study, a systematic review of CBT and mindfulness techniques in patients with psoriasis showed improvement in anxiety levels but not in depression^75^. These findings suggest that depressive symptoms may be difficult to improve without improving the underlying disease. Of note, these studies focused on patients with moderate to severe disease. Therefore, conclusions on efficacy cannot be drawn for patients with mild or moderate disease. Furthermore, there are multiple meta-analyses illustrating the efficacy of CBT in improving both anxiety and depression in children, adolescents, and adult patients^76–83^. Thus, future studies in large, diverse populations are warranted to understand the efficacy of education and CBT in treating anxiety and depression in patients with AD.

Relaxation and stress reduction techniques may have a role in the treatment of AD-related anxiety and depression, but few studies have rigorously evaluated these strategies^84,85^. PMR is a muscle relaxation therapy that has been shown effective in treating anxiety disorders, including panic and generalized anxiety disorders^86,87^. Bae, et al. found that PMR intervention consisting of physical and mental components for 4 weeks significantly reduced anxiety and depression. Several other case series, both in adults and pediatrics, have shown similar results^30,88^.

Ion exchange water softeners were shown to be effective in improving depression in AD despite lack of improvement in primary outcome of the study as measured by eczema severity measured by Six Area Six Sign Atopic Dermatitis Score (SASSAD)^51^. Decreased depression symptoms despite the absence of objective improvement in eczema might be due to perceived improvement of eczema in the setting of significant change in patient-oriented eczema scores reported in the study. Interestingly, a similar effect was not observed for anxiety in the study. This may indicate that anxiety/depression can be multifactorial in nature including diet, lifestyle, gut microbiome, and genetic influences, rather than dependent on eczema severity alone^89,90^. Further work is needed to understand this difference and how this may play into mental health and quality of life in patients with eczema.

Our results support other systematic reviews and meta-analyses on mental health interventions for patients with other dermatological diseases. A previous study found that patients taking isotretinoin for acne had significant improvement in depressive symptoms after treatment^91^. Fleming et al.^92^ demonstrated that patients with moderate-severe psoriasis had significant improvement in depression after treatment with a biologic. We hypothesized that there might be a positive correlation between the improvement in AD markers and psychological metrics that could be tested if we had access to individualized data from each trial. Among several possibilities, this correlation could be linear with a set improvement in anxiety or depression stemming from some equivalent improvement in itch or rash, or psychological improvement may be felt only after a certain threshold of improvement in itch or rash was met. Currently, available data cannot conclude whether a patient suffering from severe AD would be expected to have complete resolution of the related psychological burdens should his or her AD symptoms significantly improve. Therefore, future studies should consider releasing participant-level results so this needed analysis can be performed.

It should be noted that none of the pharmacological studies included in this systematic review evaluated anxiety and depression as a primary outcome. Future studies evaluating topical vs systemic AD treatment in improving anxiety and depression, as well as the use of anti-anxiety and/or anti-depressants in patients with severe AD would be of tremendous value. Linnet et al. indicate that patients with baseline high anxiety levels seemed to be the ones who benefited the most from non-pharmaceutical interventions. Further research is needed to identify AD patients who will benefit the most from either pharmaceutical or non-pharmaceutical interventions. Further research is also needed to understand patients’ and caregivers’ preferences for pharmacologic and non-pharmacologic interventions for AD in addition to the relative efficacy of these different approaches. Access to mental health providers, particularly those who understand AD might be a barrier for patients who prefer non-pharmacologic interventions. Given that anxiety and depression associated with AD are contextual and related to the experience of living with AD symptoms, referral to mental health providers who lack knowledge of the condition may not be helpful^93^.

The present study has other limitations. As mentioned previously, the pharmacological interventions were conducted on patients with moderate/severe AD. Since anxiety and depression are usually evaluated as a secondary outcome, there are several studies that we excluded due to incomplete data despite attempts to request data from the study investigators. Furthermore, these pharmacological studies do not preclude or determine if enrolled patients are already receiving pharmacological treatment or therapy for their anxiety and/or depression. There was some heterogeneity in the techniques and/or tools used, as well as the design of studies included in the non-pharmacological treatment as they are evaluating very disparate interventions (i.e. use of water softener vs cognitive behavioral therapy). This precludes us from conducting similar meta-analyses for non-pharmacological interventions. Overall, this work offers the first systematic review of the pharmacologic and non-pharmacologic treatments for anxiety and depression in the AD patient population. Our findings indicate that patients with moderate to severe AD may directly benefit from treatments targeting the mental health burdens related to AD, even in cases where the AD symptoms are stable.

## Conclusion

In conclusion, pharmacological interventions intended to improve AD severity in patients with moderate to severe disease are also effective in decreasing anxiety and depression. Despite the significant mental health burden of AD, there are a limited number of studies evaluating the efficacy of interventions targeting anxiety and depression in this population. This underscores the need for future RCT in large, diverse patient populations to determine whether different pharmacological and non-pharmacological interventions can improve anxiety and depression in patients with AD.

### Supplementary Information

Supplementary Information.

## Data Availability

The datasets used and/or analyzed during the current study are available from the corresponding author upon reasonable request.
