# Supplementary material for: Interventions for anxiety and depression in patients with atopic dermatitis: a systematic review and meta-analysis
Source: Sci Rep. 2024 Apr 17;14:8844. doi: 10.1038/s41598-024-59162-9 (PMC11024101; doi:10.1038/s41598-024-59162-9)
Supplement: Supplementary file 1 — Supplementary Information. [file 41598_2024_59162_MOESM1_ESM.pdf]

Supplementary Table 1 – MESH / EMTREE terms used for search strategy.

Database	MESH / EMTREE / keywords
PubMed / MEDLINE	(("Dermatitis, Atopic"[Mesh]) OR "Eczema"[Mesh]) AND ("Depression"[Mesh] OR "Anxiety"[Mesh])
EMBASE	(‘atopic dermatitis’/exp OR ‘eczema’/exp) AND (‘depression’/exp OR ‘anxiety’/exp)
PsychINFO	(Eczema OR dermatitis) AND (anxiety OR depression, major OR depression, emotion)

Search results were further filtered for human studies only, articles in English, articles available as full-texts, and full-length articles in print (removing pre-prints, news articles, conference abstracts, and conference proceedings).

Supplementary Figure 1 – Funnel plot

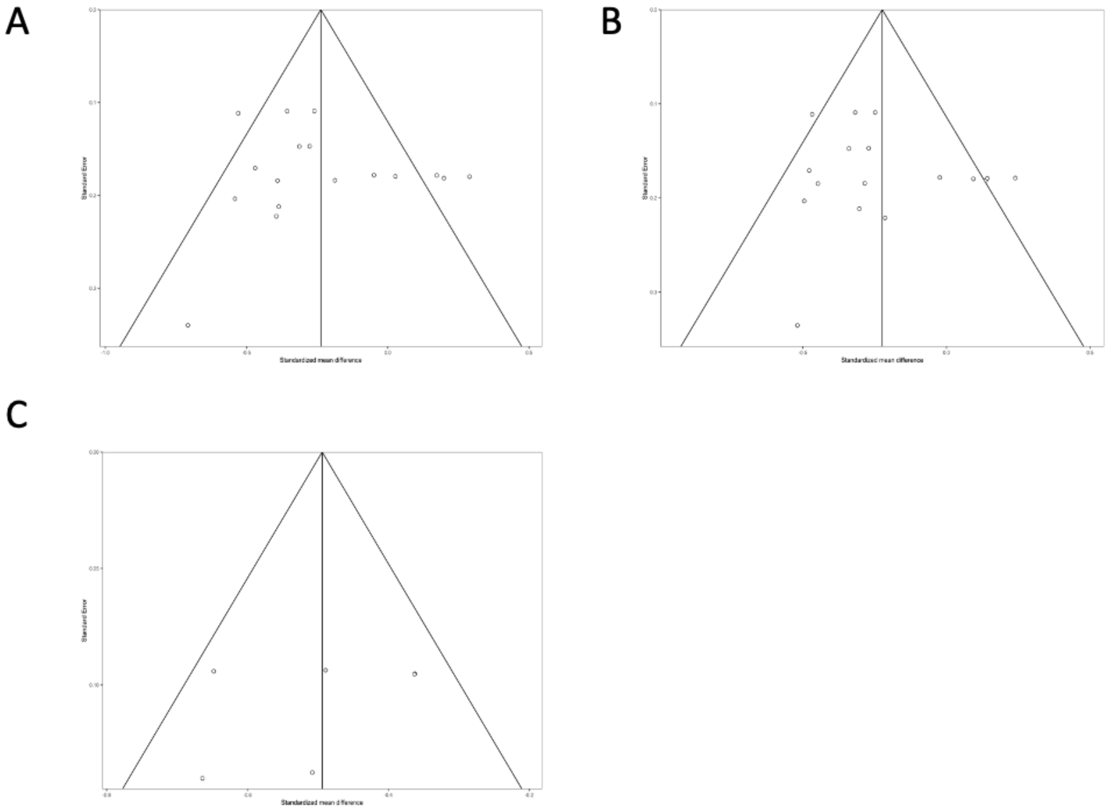

Supplementary figure 1. Funnel plot of interventions included in the meta-analysis for anxiety (A), depression (B), or both anxiety and depression (C) indicating no concern for publication bias

Supplementary Figure 2 – Sensitivity analysis

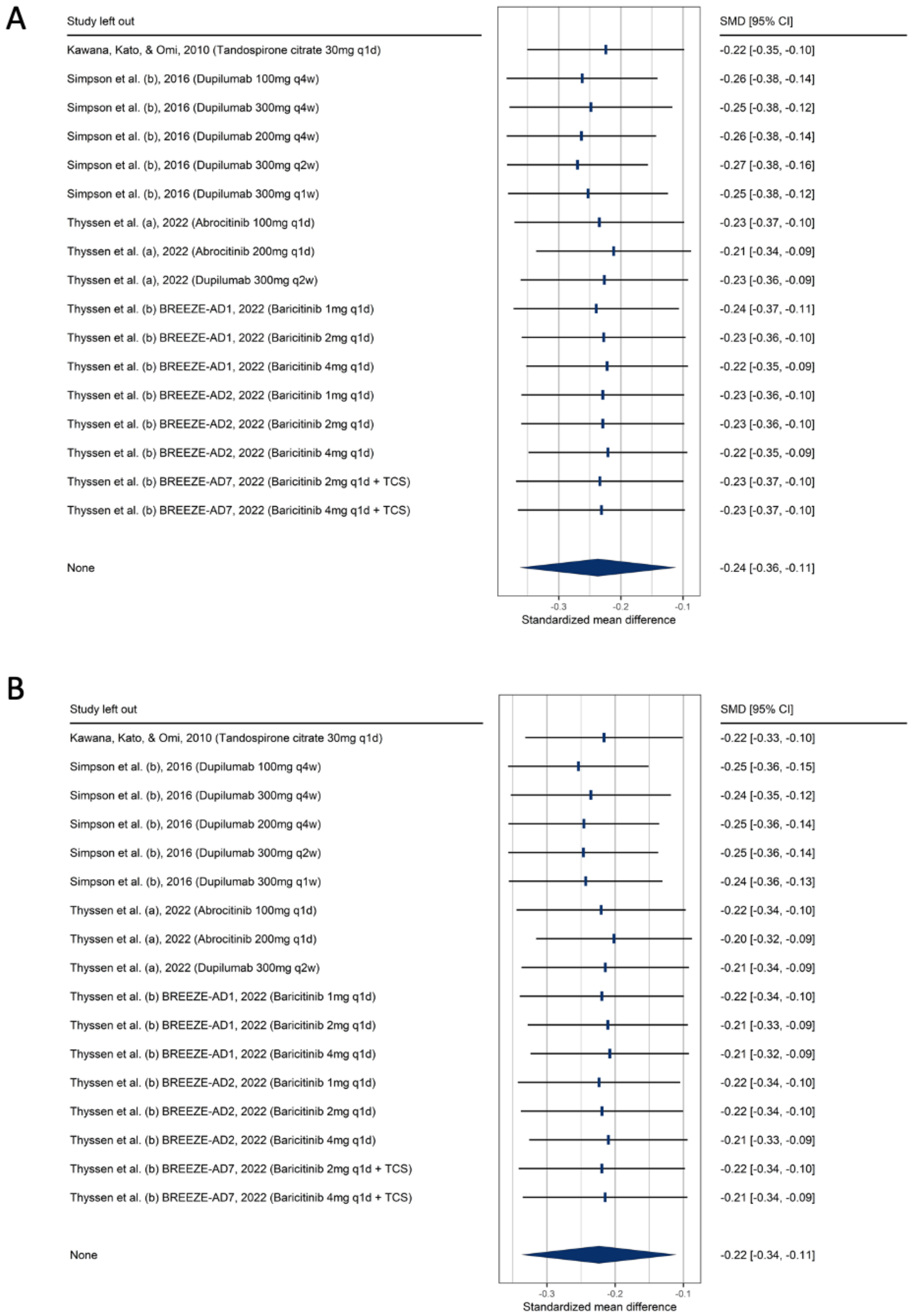

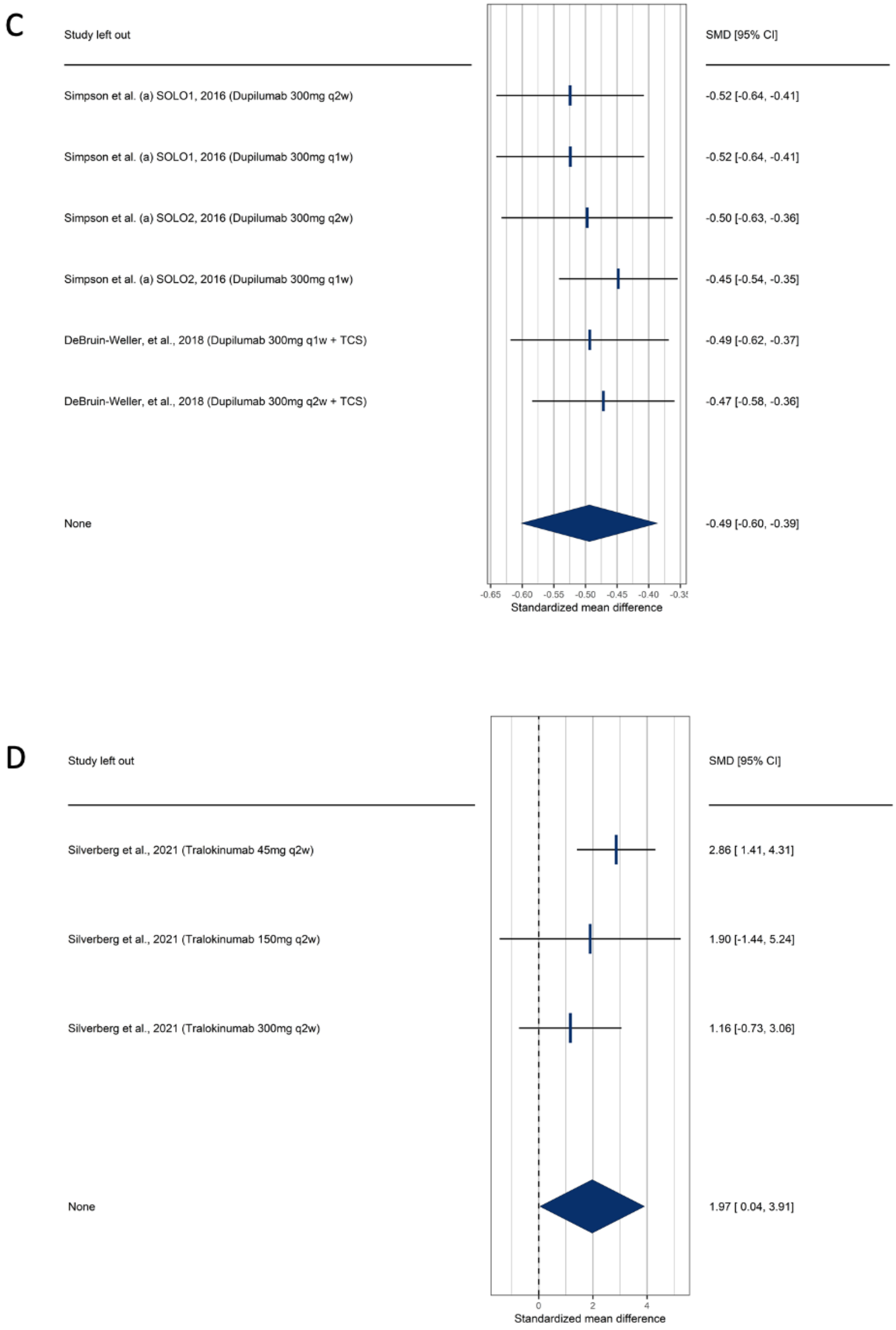

Supplementary figure 2. Sensitivity analysis showing the effect of leaving one study out for anxiety (A), depression (B), both anxiety and depression (C) as well as all the intervention arms for Silverberg et. al. No effect is observed on overall results

Supplementary Figure 3

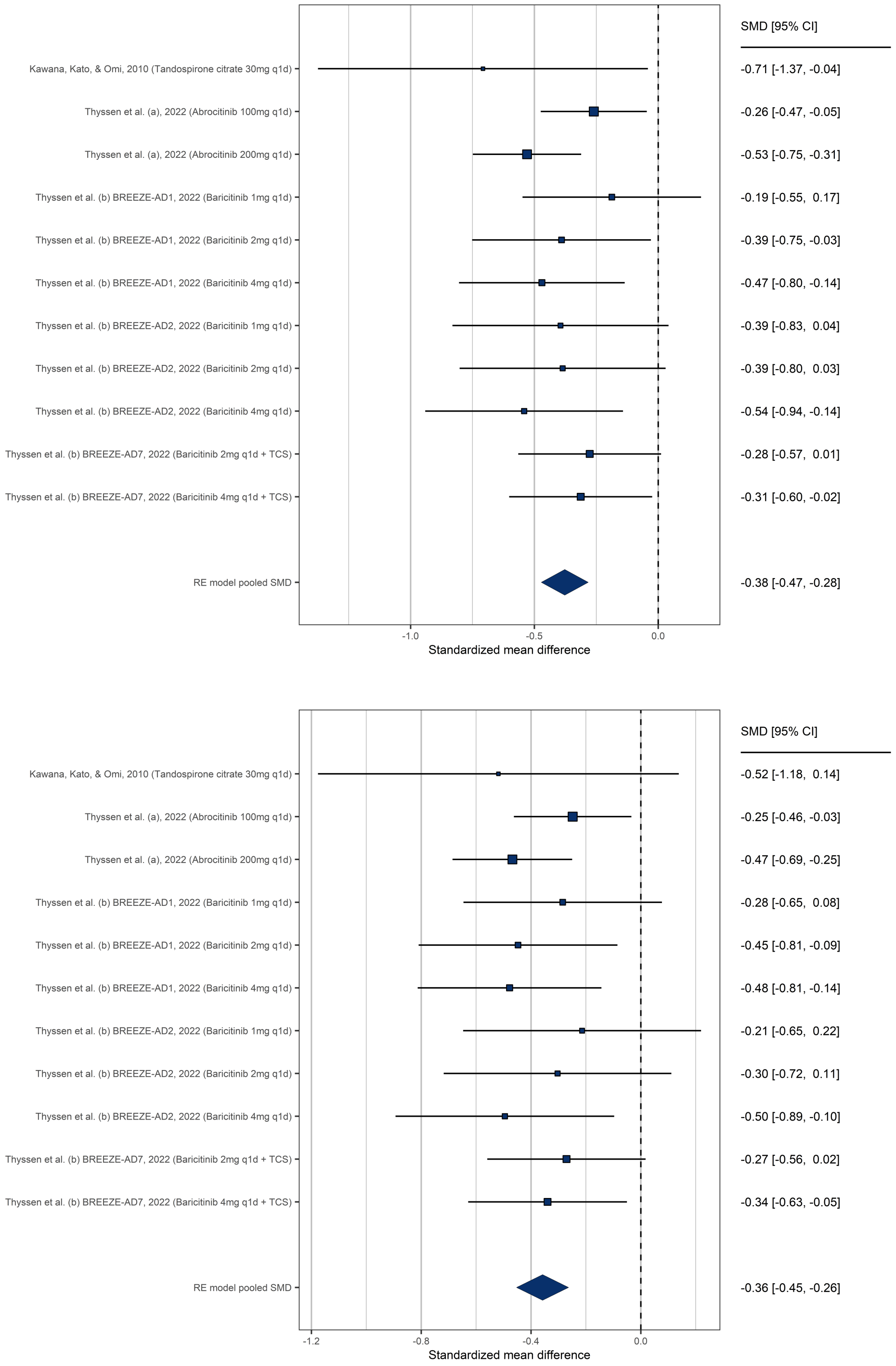

Supplementary figure 3. Forest plot for anxiety and depression of all the non-dupilumab interventions showing similar effect.
